# About the age and depositional depth of the sediments with reported bipedal footprints at Trachilos (NW Crete, Greece)

**DOI:** 10.1038/s41598-022-23296-5

**Published:** 2022-11-02

**Authors:** Willem Jan Zachariasse, Lucas J. Lourens

**Affiliations:** Department of Earth Sciences, Faculty of Geoscience, Utrecht University, Vening Meinesz building A, Princetonlaan 8a, 3584 CB Utrecht, The Netherlands

**Keywords:** Geology, Palaeomagnetism, Palaeontology, Sedimentology

## Abstract

New data on the foraminifers and the regional geological setting of the Trachilos sediments (NW Crete, Greece) from which Gierlinski et al. (Proc Geol Assoc 128: 697–710, 2017) described hominin-like footprints show that the published 6.05 Ma-shallow marine interpretation is incorrect. In our new interpretation, the Trachilos succession is Late Pliocene and part of a shallowing marine series that became subaerially exposed some 3 millions of years ago. Placed in a larger geological context, Crete was an island during the Late Pliocene and separated by ~ 100 km of open sea from the nearest European mainland, and therefore out of reach of Late Pliocene hominins.

In 2017, Gierliński and et al*.*^1^ reported hominin-like footprints from a marginal marine sediment succession along the coast at Trachilos on NW Crete and used litho- and biostratigraphic arguments to date the footprints as Messinian with an age of ~ 5.7 Ma. The authors further claim that the bipedal trackmaker came from the north via a land bridge connecting mainland Greece with Crete. The age of ~ 5.7 Ma has recently been adjusted to ~ 6.05 Ma based on new magneto- and biostratigraphic data^2^. Doubts about attributing the pictured bedding surface phenomena to footprints have been expressed in Meldrum and Sarmiento^3^ whereas Crompton^4^ acknowledges that they are footprints but questions their hominin origin. The age of ~ 6.05 Ma is surprising given the previously published data on the stratigraphy and depositional history of the Neogene in northwestern Crete^5–9^. In this study we will give an overview of the regional Neogene stratigraphy and argue why a Late Pliocene age for the Trachilos sediments fits better with the new data on the foraminifers and the geological setting.

## Regional lithostratigraphy and depositional history

**Figure 1:**
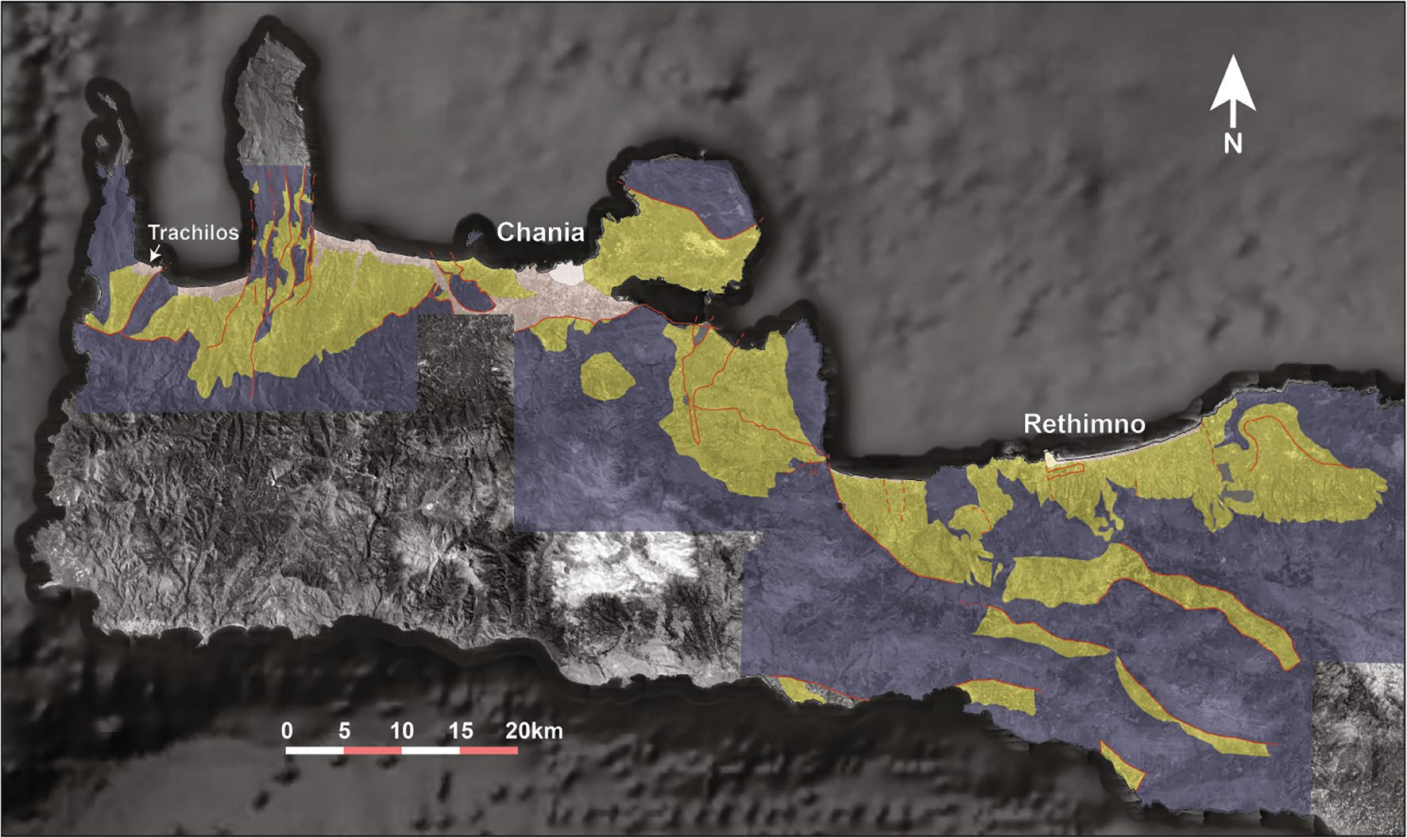
Google Earth satellite image of western Crete (imagery date 9–1-2018) with the distribution of Upper Cenozoic sediments after Freudenthal^5^ and Meulenkamp^10^. Legend: undifferentiated basement (grey); Neogene (yellow); Quaternary (rose); not mapped (no color). Location of the Trachilos site is shown by arrow.

**Figure 2:**
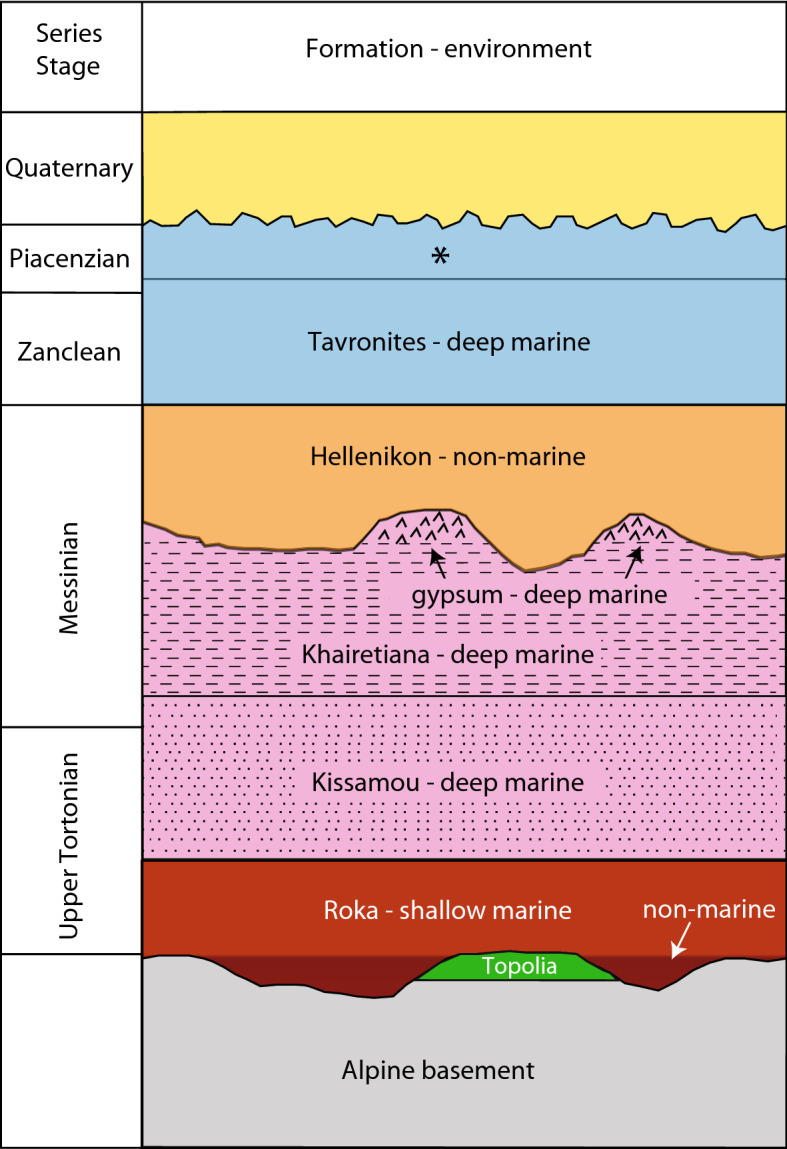
Lithostratigraphy of the Neogene in the area west of Chania after Freudenthal^5^ and Meulenkamp et al.^8^. The chronostratigraphic position and depositional environment of the lithostratigraphic units is based on Van Hinsbergen and Meulenkamp^9^ and this study. Asterisk refers to the shallowing top of the Tavronitis Fm which is nowhere exposed except probably at Trachilos.

The Neogene sediments of western Crete (Fig. 1) have been mapped, described and for the first time formally subdivided into lithostratigraphic units by Freudenthal^5^ and Meulenkamp^10^. In the area west of Chania (including the Trachilos site), Freudenthal^5^ has distinguished five units which are used to this day^6,7^. The lithostratigraphic subdivision of Meulenkamp et al.^8^ for this area includes the same units under partly different names from which we here only adopt the Hellenikon Fm. Figure 2 gives an overview of the litho- and chronostratigraphy of the Neogene in the area west of Chania. The succession in question unconformably overlies remnants of older Neogene breccio-conglomerates (described by Kopp and Richter^11^ under the name Topolia Fm) and thus belong to the basement of the stratigraphic succession depicted in Fig. 2. Basin subsidence began with the filling of basement depressions with fluvial clastic sediments followed by the area-wide deposition of shallow marine sandstones (often coarse and pebbly) and bioclastic to reefal limestones with characteristic fossils such as the larger foraminifer *Heterostegina* and *Clypeaster* echinoids. The fluvial and shallow marine deposits together represent the Roka Fm of Freudenthal^5^. Except that the shallow marine sediments overlie the depression-filling fluvial deposits, they also overlie rocks of the three nappes that make up the Alpine basement in this area with the Pindos unit being the highest structural unit and the Phyllite-Quartzite unit the lowest with the Tripolitza unit in the middle^12^. Rapid deepening of the basin to upper bathyal depths^9^ is materialized in the deposition of deep marine bluish grey mostly amorphous marls with minor sandy turbidites (Kissamou Fm)^5^. In one specific small area, the Kissamou Fm is dominated by sandy turbidites and mapped by Freudenthal^5^ as Koukounaras Fm. The Kissamou Fm belongs to the Upper Tortonian—Lower Messinian^9^. Trends in coarseness and thickness of turbiditic sandstones point to a southern hinterland during this time span^5^.

The next higher lithostratigraphic unit defined by Freudenthal^5^ is the Khairetiana Fm which is Messinian in age^13^ and deposited in an upper bathyal environment as well^9^ but unlike the Kissamou Fm, the amorphous marls of this unit are beige white and more calcareous and may alternate with brownish laminated marls (sapropels) while the turbiditic sandstones of the Kissamou Fm are often replaced by turbiditic calcarenites^5^. Gypsum in the uppermost part of this deep marine unit accumulated during the early phase of the Messinian Salinity Crisis (MSC), which began in the eastern Mediterranean at 6.00 Ma^14^.

Subaerial erosion during the subsequent phase of desiccation of the Mediterranean has removed most of the gypsum. The truncation horizon is overlain by an up to 200 m thick unit of non-marine reddish conglomerates and fines of the uppermost Messinian (Hellenikon Fm) ^8^ and that unit is again overlain by the Pliocene Tavronitis Fm^5^. The latter formation is marine and composed of whitish calcareous marls (Trubi facies) in the basal part and replaced upwards by less calcareous greyish marls with many gravity deposits and slumps.

Sediments of the Tavronitis Fm belong to the Zanclean and lowermost Piacenzian^6,9^. The estimated paleodepth of ~ 600 m for these sediments^9,15^ implies a similar amount of uplift for this part of Crete since the Piacenzian. An upwards shallowing trend, however, has not been reported but since the regional basin fill is tilted north^5,8^, the material record of shallowing and final emergence is probably buried below the northern coastal plain and adjacent sea floor (see Fig. 2). The emergence of central Crete is dated at ~ 3 Ma^15^.

The Upper Neogene basin fill in the area west of Chania (Fig. 1) is dissected by a system of roughly N-S and E-W trending normal faults^5,12,16^. These faults were inactive during sedimentation because all lithostratigraphic units are easily recognizable from the west coast up to Chania^5^. For example, the lithology and depositional depth of the three deep marine lithostratigraphic units in Fig. 2 remain the same across the N-S trending blocks of uplifted basement^9^. Also, the current southern basin boundary consists of young N-S and E-W normal faults. Remnants of basin sediments south of this boundary indicate that the actual basin bounding fault which accommodated some 650 m of basin sediments was located farther to the south well into the basement (WJZ, unpublished data).

### The geology of the Trachilos area

**Figure 3:**
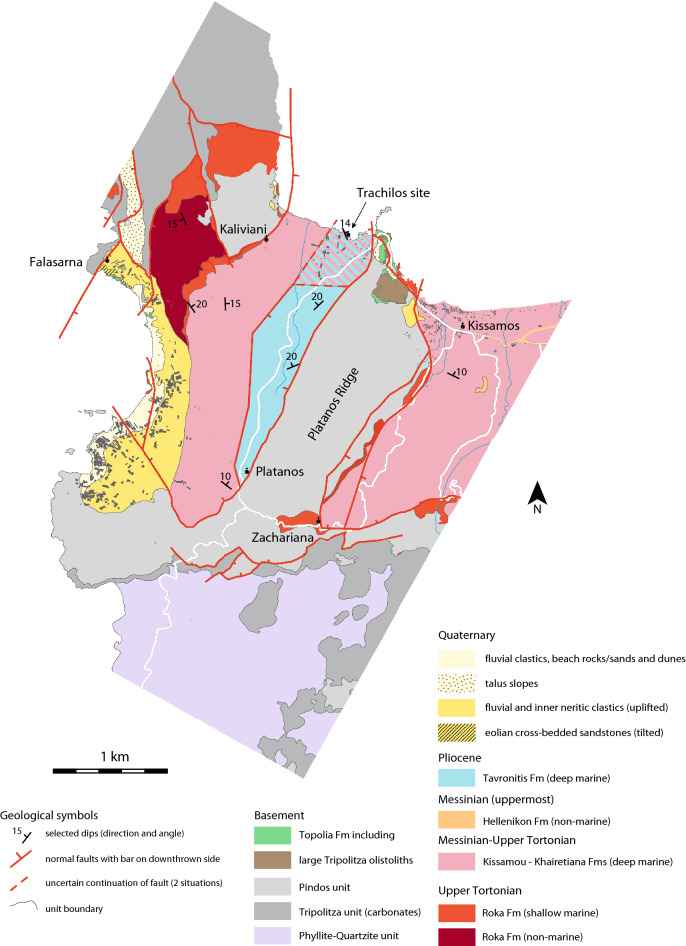
Geological map of the greater area around the Trachilos site (this study). Original map was digitized on Google Earth and based on a large number of GPS fixed field observations. The map shown is constructed from the data digitized in Google Earth and projected on OpenStreetMap topographical data using MaPublisher software (https://www.avenza.com/mapublisher/). Pink/blue shading encloses the area of Pliocene (blue) or Messinian deposits (pink) dependent on the fault geometry (see text).

Published geological maps of the area around the Trachilos site are based on the official geological map of Greece^17,18^ (see map of Mountrakis et al. ^19^) or Freudenthal^5^ (see Frydas^6^ and Kontopoulos et al.^20^). Only the one in Frydas and Keupp^7^ is based on own (student) fieldwork but lacks faults and any further reference. Figure 3 presents our new geological map of the area based on fieldwork conducted over a much wider area by WJZ in 2019 and 2021. The coastal plains in the area were formed by coastal erosion after basin emergence, faulting, overall northward tilting, and under ongoing uplift. In Fig. 3, the cultivated northern coastal plain is stripped of any Quaternary sediments except the reddish fluvial conglomerates in a single inland outcrop and beach rocks of cemented sandstones and conglomerates along the coast. Quaternary fluvial conglomerates at heights between 20 and 40 m immediately west of Kissamos proves the ongoing uplift of this part of Crete. At Falasarna, the uplifted coastal plain with Quaternary fluvial conglomerates/fines, inner neritic sandstones/gravels and eolian cross-bedded sandstones allows a similar conclusion (see Fig. 3).

The geological map in Fig. 3 further shows that the Neogene basin fill is interrupted by a NE-SW trending horst block of Pindos rocks (called here the Platanos Ridge). The Neogene succession is similar on either side and starts with dominant fluvial conglomerates filling basement depressions (mapped as the non-marine part of the Upper Tortonian Roka Fm if they are thick). Marine subsidence is materialized by bioturbated sandstones locally rich in *Heterostegina* (representing the shallow marine part of the Upper Tortonian Roka Fm). These shallow marine sediments overlie the fluvial unit as well as rocks of the Pindos and Tripolitza basement units (Fig. 3). Bluish grey silty marls of the Kissamou Fm and beige white calcareous marls with turbiditic calcarenites of the Khairetiana Fm are poorly exposed and mapped as one single Messinian-Upper Tortonian unit. This unit is, as elsewhere in the area west of Chania^7,9^, deep marine (see also Supplementary Materials). A small erosional remnant of the fluvial uppermost Messinian (Hellenikon Fm) is preserved east of the Platanos Ridge. Pliocene sediments (Tavronitis Fm) occur directly west of the Platanos Ridge and consists of (poorly exposed) beige amorphous and brownish laminated marls (sapropels) with turbiditic bioclastic calcarenites and occasional slump. Burrows are common. Some calcarenites have a gravelly base with bivalve debris whereas others show planar laminations (Freudenthal^5^; this study). These sediments are deep marine^9^. Foraminiferal associations from an outcrop along the road to Platanos (3 km south of the Trachilos site) point to an age of 3.60–3.57 Ma (earliest Piacenzian) and a depositional depth of 500–750 m (see in Supplementary Materials). Calcareous nannoplankton associations from nearby locations provide a latest Zanclean-earliest Piacenzian age as well^6,20^. Apparently, a normal fault with a large offset juxtaposed deep marine Pliocene against deep marine Messinian leaving the deep marine evaporites (if preserved), the uppermost Messinian and Lowermost Pliocene buried in the hanging wall block. In which direction this fault will continue below the coastal plain is uncertain.

### The Trachilos site

**Figure 4:**
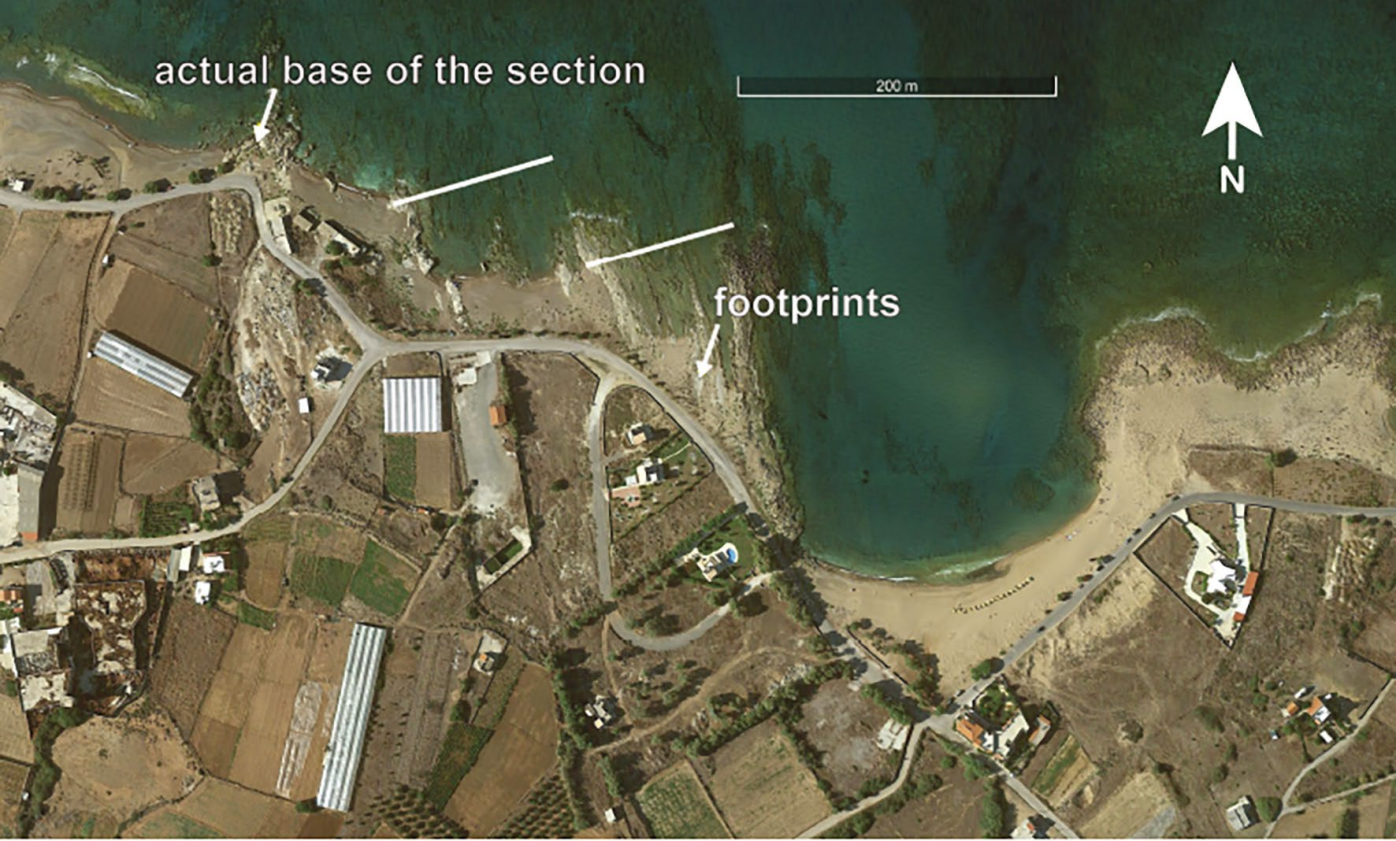
Google Earth satellite image of the Trachilos site (imagery date 9–1-2018). White lines mark the section as published in Gierlinski et al.^1^ and Kirscher et al.^2^.

**Figure 5:**
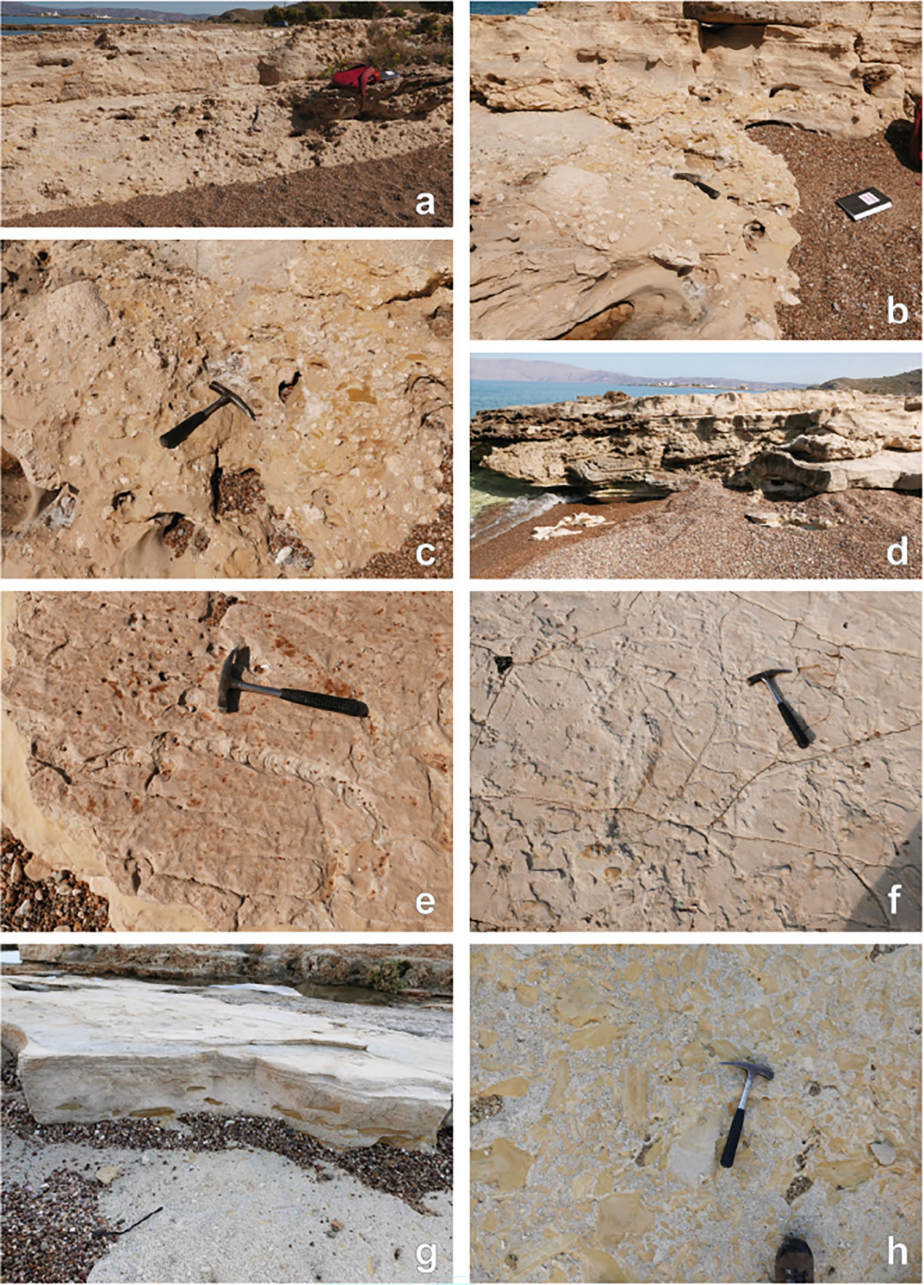
Pictures of the Trachilos sediments: (**a–c**) debris flow deposit of silty marls, calcarenites and algal nodules; (**d**) debris flow deposit between slumped (below) and in situ turbiditic calcarenites (above); (**e–f**) burrows on top of fine calcarenites; (**g**) silty marl pebbles/flakes (with sample C) in the coarse calcarenite just below the footprint layer.

The published section is ~ 40 m thick (based on an average dip direction and angle of 70°/14°) with the upper 20–25 m (including the footprints) being the best exposed part (Fig. 4). The section, however, continues a little further west along the coast extending the published section downwards by another 20 m so that the total stratigraphic thickness is ~ 60 m including several non-exposed intervals (Fig. 4). The sediments are dominated by well cemented calcarenites rich in marine skeletal debris from, among others, the red coralline alga *Lithothamnion*, bryozoans, and bivalves. Most calcarenites show planar laminations while some others change laterally in thickness or show normal grading indicating a turbiditic origin for the calcarenites. A few debris flow deposits are characterized by mixtures of grey brownish silty marls, coarse calcarenites, and *Lithothamnion* nodules (Fig. 5a–c). At one point such a debris flow overlies a slumped package of calcarenites (Fig. 5d). Large burrows are common. The horizontal burrows shown in Fig. 5e–f are several tens of centimeters long and several centimeters wide.

The section exposes only a few interbeds of poorly cemented grey brownish silty marls but more often such silty marls occur as pebbles and flakes in the coarse calcarenites (Fig. 5g–h). Three samples from these silty marls were subjected to a micropaleontological study. Sample A consists of silty marls with sub centimeter thick calcareous siltstones obtained from three different levels in the uppermost part of the section. Sample B comprises several silty marl pebbles/flakes from the 30 cm thick coarse calcarenite immediately below the footprint bed. Sample C is from a silty marl bed at the base of the published section. Samples were soaked in water for one week and then washed over two sieves with mesh sizes of 595 and 125 micron after which the dried 125–595-micron fractions were studied for their microfossil content.

### Washed residues and microfossils

Residues are dominated by lime fragments (partially crystalline) representing the calcareous cement. Microfossils are cement-encrusted and very poorly preserved. The identified foraminifers in the three samples are listed below where frequencies are estimates rather than based on counting.

Sample A: Overall composition: echinoid spines, bryozoan fragments, ostracods, foraminifers, and fish debris are common. The benthic foraminiferal association consists of *Elphidium crispum* (common), *Cibicides lobatulus-refulgens* (rare), *Hanzawaia boueana* (rare), *Asterigerina planorbis* (common), *Rosalina* sp*.* cf. *R. globularis* (rare), *Ammonia parkinsoniana* (rare), *Bolivina plicatella* (trace), non-costate *Bulimina* (trace), *Bolivina spathulata* (common) and *Rectuvigerina bononiensis* (common). The planktonic foraminiferal association consists of *Turborotalita quinqueloba* (common).

Sample B: Overall composition: echinoid spines, bryozoan fragments, ostracods, and foraminifers are common. Probably a few fish debris. The benthic foraminiferal fauna consists of *E. crispum* (common), *E. fichtelianum* (rare), *C. lobatulus-refulgens* (rare), *H. boueana* (trace), *A. planorbis* (common), *R.* sp*.* cf. *R. globularis* (rare), *B. plicatella* (trace), miliolids (rare), non-costate *Bulimina* (trace), *B. spathulata* (rare-common) and *R. bononiensis* (common). The planktonic foraminiferal association is made up of *Orbulina universa* (common) and the *Globigerina bulloides* group (common and mainly the *pseudobesa* type).

Sample C: Overall composition: echinoid spines, ostracods, foraminifers are common; fish and bryozoan debris are rare. The benthic foraminiferal association consists of *E. crispum* (common), *E. fichtelianum* (rare), *C. lobatulus-refulgens* (common), *H. boueana* (common), *A. planorbis* (common), *R.* sp*.* cf. *R. globularis* (rare), *B. plicatella* (trace), *Nonion boueanum* (rare), *A. parkinsoniana* (rare), non-costate *Bulimina* (trace), *B. spathulata* (common) and *R*. *bononiensis* (rare-common). The planktonic foraminiferal association consists of the *G. bulloides* group (common and mainly the *pseudobesa* type), *O. universa* (trace) and cf. *Globigerinoides obliquus* (trace).

### Benthic foraminiferal associations

The benthic foraminiferal associations in the three Trachilos samples are remarkably similar and composed of two groups of taxa each occupying different habitats. The first group of *R. bononiensis, B. spathulata* and non-costate *Bulimina* is infaunal and often numerous in environments with increased organic carbon input and shallow in-sediment oxygen penetration such as the shallow marine part of the Rhone prodelta^21^ with the note that the extinct *R. bononiensis* is represented here by *Rectuvigerina phlegeri*. Relatively high numbers of these taxa are also reported from upper bathyal, organic-rich laminated sediments (sapropels) in the Upper Neogene of Crete and Gavdos^22–25^. These laminated sediments formed under dysoxic bottom water conditions and elevated organic carbon input because of diminished rates of deep-water formation and increased surface water productivity at times of maximum northern summer insolation and associated wet climatic conditions^26^. Late Quaternary sapropels in the open eastern Mediterranean formed below water depths of 300 m (Rohling and Gieskes^27^) and the same minimum depth is concluded for the Upper Neogene sapropels of Crete and Gavdos^9,25,28^.

The taxa of the second group are epifaunal and attached and live in well-oxygenated and oligotrophic shallow normal marine environments^29–31^ where *E. crispum* and *C. lobatulus-refulgens* have been shown to be epiphytes^32^ while *B. plicatella, H. boueana, A. planorbis* and *R.* sp*.* cf. *R. globularis* are suspected to be epiphytes^30,31,33^.

### Depositional depth of the Trachilos sediments

The presence of two ecologically separate groups of benthic foraminifers in the Trachilos samples raises the question to what extent the associations are in situ. The possibility that all benthic foraminifers are reworked from older sediments is not likely because composition and preservation is similar within and between samples. If the associations lived simultaneously but were partly displaced, then the shallow dwelling epifaunal and attached species should be ex situ because they are, and especially those clinging to hard substrates or attached to plants, most prone to displacement by storms. Benthic foraminiferal associations described from Upper Neogene sapropels of Crete and Gavdos are commonly represented by in situ bolivinids, non-costate buliminids and rectouvigerinids with variable numbers of displaced shallow dwelling epifaunal and attached species^22,25^. Such upper bathyal sapropel associations are thus remarkably similar to the Trachilos associations thereby suggesting a deep marine origin of the Trachilos sediments. Alternatively, if the Trachilos associations are entirely in situ then they indicate a shallow marine setting with muddy bottoms for infaunal species within a short distance of (vegetated) sand or gravel substrates for attached benthic foraminiferal species (e.g., the Rhone prodelta example in the previous section).

A first minus for the deep marine interpretation (the sapropel-case) is that sapropels typically consist of organic-rich laminated marls but such marls are absent in the Trachilos succession. A second minus is the absence of benthic foraminiferal depth marker species, such as the ones defined for water depths exceeding 100 m in Van Hinsbergen et al.^34^. The absence of these depth markers in the Trachilos samples would be in support of the shallow marine interpretation unless the published age model for the Trachilos site is correct. If it were true that the Trachilos sediments are ~ 6.05 million years old^2^ then the absence of benthic foraminiferal depth markers has nothing to do with water depth but with the vanishing of this group from the Mediterranean some 6.7 million years ago^25,35^, possibly in response of an increase in the salinity of the Mediterranean towards hypersaline values^14^.

Other depth indicators to discriminate between the deep marine (sapropel-case) and shallow marine interpretation are absent or not applicable. For example, a reliable method to reconstruct the depositional depth of marine sediments is the ratio planktonic to benthic foraminifers^36^, but this method is useless here because of the extremely poor preservation of the foraminifers. Also, the planktonic foraminiferal composition does not supply any specific information on water depth since thermocline dwellers such as neogloboquadrinids and globorotaliids are absent in our samples. The mere presence of planktonic foraminifera suggests a minimum depth of ~ 50 m for the Trachilos sediments because surface sediment samples in the Adriatic Sea are almost barren in planktonic foraminifers above that depth^37^. Also, the burrows shown in Fig. 5e-f do not supply a clue about the depositional depth of the Trachilos sediments. The burrow in Fig. 5e resembles *Bichordites monastiriensis* which is attributed to a spatangoid echinoid^38^. The burrows in Fig. 5f resemble *Sinusichus sinuosum* in Belaustegui et al.^39^. Thalassinoides mentioned in Gierlinski et al.^1^ is probably a different name for the same burrow. Both Bernardi et al. ^38^ and Belaustegui et al.^39^ described their trace fossils from shallow marine sediments, but they occur in deep marine environments as well^40^. The sediments themselves also give no indication of the depositional depth except that the absence of sapropels argues for a setting < 300 m. The pictured ripples with so-called wrinkled crests (see Fig. 3c in Gierlinski et al.^1^) are interpreted as microbial mat-related structures indicative of a marginal marine environment but without providing geochemical or biomarker evidence for this interpretation. The pictured ripples themselves are either wave or current ripples. The turbiditic calcarenites and debris flow deposits (this study; Fig. 5) may have deposited on a shallow marine slope but just as well in a deep marine setting. Why the layer of matrix supported unsorted bioclasts in Fig. 5a is interpreted as tsunamite (see Fig. 3b in Gierlinski et al.^1^) and not as a submarine debris flow deposit (this study) has not been explained by Gierlinski et al.^1^.

The conclusion of the above discussion is that the Trachilos fossils and sediments do not allow to discriminate between a shallow versus deep marine setting provided the Kirscher et al.^2^ age model is correct. In an older, i.e., pre-6.7 Ma (see above) or younger (Pliocene) age model, the absence of benthic foraminiferal depth markers would be a convincing case for a shallow (< 100 m) marine setting.

### The age of the Trachilos sediments

Figure 3 shows that the NE-SW trending segment of the normal fault that juxtaposed Pliocene against Upper Tortonian-Messinian sediments may continue below the coastal plain in a northeasterly direction or changes direction more eastwards so that the Trachilos sediments belong either to the Pliocene or to the Upper Tortonian-Messinian (shown as pink/blue shading in Fig. 3). Normally, planktonic foraminifers should discriminate between an Upper Tortonian-Messinian or Pliocene age but the rare and poorly preserved representatives of *Turborotalita quinqueloba* (sample A) and the *Globigerina bulloides* group and *Orbulina* (samples B and C) do not allow a solid age determination. *Neogloboquadrina acostaensis* and *N. atlantica* are absent not only in our samples but also in those of Gierlinski et al.^1^. Scanning electron microscope (SEM) pictured specimens of both species (see Fig. 5 in Gierlinski et al.^1^) belong to *Globigerina pseudobesa* because they are spinose unlike the non-spinose, cancellate wall texture of neogloboquadrinids. For the same reason are the figured specimens of *Neogloboquadrina acostaensis* (see Fig. S6 in Kirscher et al.^2^) assignable to *Globigerina pseudobesa*. Furthermore, SEM pictures of *Sphaeroidinellopsis multiloba* (see Fig. 5 in Gierlinski et al.^1^) show no cortex and belong to *Orbulina* with an incompletely embracing final chamber whereas the pictured *Turborotalita multiloba* (see Fig. S6 in Kirscher et al.^2^) seems indistinguishable from *T. quinqueloba*. The planktonic foraminiferal associations described by Gierlinski et al.^1^, Kirscher et al.^2^ and in this study thus is composed of some five long-ranging surface-dwelling species with no more time significance than Late Cenozoic. The same applies for the few dinoflagellate cysts in an extra sample of greenish brown marls with sub centimeter thick calcareous siltstones from ~ 1 m below the footprint layer.

Here below we will explore two different age models for the Trachilos sediments. In the first model, the five surface-dwelling planktonic foraminiferal species represent an impoverished association known from the period between 6.7 and 6.0 Ma when Mediterranean salinities had risen to hypersaline values^14^. This age interpretation of the planktonic foraminifers would not contradict the correlation of the Trachilos normal polarities to Chron C3An.1n by Kirscher et al.^2^. An age range of 6.7–6.0 Ma for the Trachilos sediments requires that the fault discussed above encloses the Pliocene south of the Trachilos site (see Fig. 3) but implies that sediments and benthic foraminifers deposited in a deep marine setting because, as discussed above, all Messinian sediments in the region are deep marine (except those belonging to the Hellenikon Fm, see Fig. 2). The 6.05 Ma-shallow marine interpretation for the Trachilos sediments as published in Gierlinski et al.^1^ and Kirscher et al.^2^ is therefore demonstrably incorrect despite the absence of benthic foraminiferal depth marker species (see discussion in previous section). A 6.7–6.0 Ma age and deep marine setting for the Trachilos sediments is also not very likely given the lack of sapropels and the abundance of shallow marine fossils (benthic foraminifers and (debris of) bryozoans, coralline algae and bivalves), which, though ex situ due to the deep marine setting in this age model, should have lived in a normal marine coastal environment while hypersaline conditions prevailed in the Mediterranean during this time span^14^.

The second age model assumes that the aforementioned fault continues in the same direction below the coastal plain whereby the Trachilos sediments belong to the Pliocene (see Fig. 3). A Pliocene age requires that the Trachilos sediments are deep marine because all Pliocene sediments in northwestern Crete are reported to be deep marine^9,15^. The absence of sapropels and benthic foraminiferal depth markers in the Trachilos sediments and their presence in deep marine (500–750 m) lowermost Piacenzian sediments along the road to Platanos (see Supplementary Materials) excludes a Pliocene age older than the earliest Piacenzian (3.60–3.57 Ma, see Supplementary Information) for the Trachilos sediments.

A Pliocene age can only be true if the Trachilos sediments belong to a younger shallowing-upward part of the Piacenzian that ends with subaerial exposure (dated at ~ 3 Ma for central Crete^15^). This shallowing sequence is not described from northwestern Crete but may outcrop in the Trachilos erosional window. The absence of benthic foraminiferal depth markers and the rare surface-dwelling planktonic foraminifers fit in this interpretation. The absence of the post-Miocene surface dwelling species *Globigerinoides ruber* can be explained by the overall scarcity of planktonic foraminifers. The Late Pliocene-shallow marine interpretation for the Trachilos site is further endorsed by lithological similarities between the shallow marine Trachilos sediments and the deep marine uppermost Zanclean-lowermost Piacenzian sediments south of Trachilos. Both show many and large burrows, turbiditic calcarenites rich in marine skeletal debris and slumps. The normal polarities measured by Kirscher et al.^2^ also fit in the Late Pliocene age interpretation and may correlate with the oldest of the three normal polarity intervals of Chron C2An between 3.6 and 3.1 Ma or even the two oldest normal polarity intervals if the non-exposed 8 m in the lower part of the published section would correlate to C2An.2r^41^.

## Discussion and conclusions

If we weigh all arguments pro and con the 6.7–6.0 Ma and the Pliocene age model then the Late Pliocene-shallow marine interpretation is clearly the most likely interpretation for the Trachilos sediments. In this age-depth model turbiditic calcarenites and debris flows deposited on a shallow marine slope.

The new age of Late Pliocene for the Trachilos sediments has implications for the claim that these sediments were walked by a bipedal trackmaker. Firstly, the shallow marine setting does not seem inviting for being traversed by hominins or any other bipedal primate but in case they did, wave action must have erased them unless the tracks were made at the high tide line during a low sea level stand at the climax of one of the glacial periods that occurred every 41 kyr in this time span (e.g., Lourens et al.^42^). Secondly, Crete was an island since the Late Tortonian but the present outline and geographic setting dates from the Late Pliocene with the deep Levantine Basin to the south and the marine South Aegean Basin (SAB) to the north^9,43^. The latter basin formed in the Late Miocene by slab roll-back driven extension of the Aegean lithosphere^44,45^ and was ~ 1000 m deep north of Crete (DSDP Site 378) during the Late Pliocene^46^. The Late Pliocene SAB separated Crete from mainland Greece and Turkey by stretches of deep water of minimally 100 km wide according to the time–space reconstructions of the Aegean lithosphere in Van Hinsbergen and Schmid^45^. Occurrences of deep marine Late Pliocene sediments on the islands of Kythira in between Crete and the Peloponnese, and Karpathos in between Crete and Turkey^15,47^ prove that these islands could not have been used as steppingstones for biped dispersal, let alone that they have been part of land bridges. Rhodes Island, on the other hand, was above sea level during the Pliocene^48^. The improbability that Late Pliocene hominins were able to sail across 100 km open sea from the nearest European mainland to Crete therefore raises questions such as who made the ichnites and are they ichnites at all. These questions call for a re-investigation of the bedding surface phenomena described as hominin-like footprints.

## Supplementary Information

Supplementary Information.

## Data Availability

All data generated or analysed during this study are included in this published article [and its supplementary information files].

## References

[1] Gierliński GD (2017). Possible hominin footprints from the late Miocene (c.5.7 Ma) of Crete?. Proc. Geol. Assoc..

[2] Kirscher U (2021). Age constraints for the Trachilos footprints from Crete. Sci. Rep..

[3] Meldrum J, Sarmiento E (2018). Comments on possible Miocene hominin footprints. Proc. Geol. Assoc..

[4] Crompton RH (2017). Making the case for possible hominin footprints from the Late Miocene (c. 5.7 Ma) of Crete?. Proc. Geol. Assoc..

[5] Freudenthal T (1969). Stratigraphy of Neogene deposits in the Khania Province, Crete, with special reference to foraminifera of the family Planorbulinidae and the genus Heterostegina. Utrecht Micropaleontol. Bulletins.

[6] Frydas D (1993). Stratigraphie du Néogène de la Crète ouest (Grèce) à l’aide de Silicoflagellés et des nannofossiles calcaires. Rev. Micropaléontol..

[7] Frydas D, Keupp H (1996). Biostratigraphical results in Late Neogene deposits of NW Crete, Greece, based on calcareous nannofossils. Berliner Geowissenschaftliche Abhandlungen.

[8] Meulenkamp, J. E., Jonkers, H. A. & Spaak, P. Late Miocene to Early Pliocene development of Crete. In *Proceedings of the Sixth Colloquium on the Geology of the Aegean Region,* 137–149 (1979).

[9] van Hinsbergen DJ, Meulenkamp JE (2006). Neogene supradetachment basin development on Crete (Greece) during exhumation of the South Aegean core complex. Basin Res..

[10] Meulenkamp JE (1969). Stratigraphy of Neogene deposits in the Rethymnon Province, Crete, with special reference to the phylogeny of uniserial Uvigerina from the Mediterranean region. Utrecht Micropaleontol. Bulletins.

[11] Kopp K, Richter D (1983). Synorogenetische Schuttbildungen und die Eigenständigkeit der Phyllit-Gruppe auf Kreta. N. Jb. Geol. Paläont. Abh..

[12] Creutzburg, N., Drooger, C. & Meulenkamp, J. in *Crete Island (1:200,000)* Vol. 1 (ed Institute of Geological and Mineral Exploration (IGMR), Institute of Geological and Mineral Exploration (IGMR), Athens, 1977).

[13] Zachariasse WJ (1975). Planktonic foraminiferal biostratigraphy of the Late Neogene of Crete (Greece). Utrecht Micropaleontological Bulletins.

[14] Zachariasse, W. J. & Lourens, L. J. The Messinian on Gavdos (Greece) and the status of currently used ages for the onset of the MSC and gypsum precipitation. *Newsletters on Stratigraphy* Published online November 2021 (2021).

[15] Zachariasse W, Van Hinsbergen D, Fortuin A (2008). Mass wasting and uplift on Crete and Karpathos during the early Pliocene related to initiation of south Aegean left-lateral, strike-slip tectonics. Geol. Soc. Am. Bull..

[16] Meulenkamp JE (1979). Lithostratigraphy and relative chronostratigraphic position of the sections Apostoli and Potamidha 1 and 2. Utrecht Micropaleontol. Bulletins.

[17] Geological Map of Greece (1:50,000), Sheet Kastelli. *Institute of Geological and Mining Research (IGME)* (1968).

[18] Geological Map of Greece (1:50,000), Sheet Paleochora. *Institute of Geological and Mining Research (IGME)* (2002).

[19] Mountrakis D (2012). Neotectonic study of Western Crete and implications for seismic hazard assessment. J. Virtual Explor..

[20] Kontopoulos N, Zelilidis A, Frydas D (1996). Late Neogene sedimentary and tectonostratigraphic evolution of northwestern Crete island, Greece. Neues Jahrbuch für Geologie und Paläontologie-Abhandlungen.

[21] Mojtahid M (2009). Spatial distribution of live benthic foraminifera in the Rhône prodelta: Faunal response to a continental–marine organic matter gradient. Mar. Micropaleontol..

[22] Jonkers HA (1984). Pliocene benthonic foraminifera from homogeneous and laminated marls on Crete. Utrecht Micropaleontol. Bulletins.

[23] Nijenhuis I (1996). On the origin of upper Miocene sapropelites: A case study from the Faneromeni section, Crete (Greece). Paleoceanography.

[24] Schenau S (1999). Organic-rich layers in the Metochia section (Gavdos, Greece): Evidence for a single mechanism of sapropel formation during the past 10 My. Mar. Geol..

[25] Seidenkrantz M-S, Kouwenhoven T, Jorissen F, Shackleton N, Van der Zwaan G (2000). Benthic foraminifera as indicators of changing Mediterranean-Atlantic water exchange in the late Miocene. Mar. Geol..

[26] Rohling EJ, Marino G, Grant KM (2015). Mediterranean climate and oceanography, and the periodic development of anoxic events (sapropels). Earth Sci. Rev..

[27] Rohling EJ, Gieskes WW (1989). Late Quaternary changes in Mediterranean intermediate water density and formation rate. Paleoceanography.

[28] Zachariasse WJ, Kontakiotis G, Lourens LJ, Antonarakou A (2021). The Messinian of Agios Myron (Crete, Greece): A key to better understanding of diatomite formation on Gavdos (South of Crete). Palaeogeogr. Palaeoclimatol. Palaeoecol..

[29] Hart M, Molina G, Smart C, Hall-Spencer J (2017). The distribution of foraminifera in the Fal Estuary (Cornwall). Geosci. South-West Engl..

[30] Jorissen FJ (1987). The distribution of benthic foraminifera in the Adriatic Sea. Mar. Micropaleontol..

[31] Murray JW (2006). Ecology and Applications of Benthic Foraminifera.

[32] Sadri S, Hart M, Smart C (2011). Foraminifera from the sea grass communities of the proposed Marine conservation zone in Tor Bay. Geosci. South-West Engl..

[33] Van der Zwaan GJ (1983). Quantitative analyses and the reconstruction of benthic foraminiferal communities. Utrecht Micropaleontol. Bulletins.

[34] Van Hinsbergen D, Kouwenhoven T, Van der Zwaan G (2005). Paleobathymetry in the backstripping procedure: Correction for oxygenation effects on depth estimates. Palaeogeogr. Palaeoclimatol. Palaeoecol..

[35] Kouwenhoven T, Seidenkrantz M-S, Van der Zwaan G (1999). Deep-water changes: The near-synchronous disappearance of a group of benthic foraminifera from the late Miocene Mediterranean. Palaeogeogr. Palaeoclimatol. Palaeoecol..

[36] Van der Zwaan G, Jorissen F, De Stigter H (1990). The depth dependency of planktonic/benthic foraminiferal ratios: Constraints and applications. Mar. Geol..

[37] Jorissen FJ (1988). Benthic foraminifera from the Adriatic Sea: Principles of phenotypic variation. Utrecht Micropaleontological Bulletins.

[38] Bernardi M, Boschele S, Ferretti P, Avanzini M (2010). Echinoid burrow Bichordites monastiriensis from the Oligocene of NE Italy. Acta Palaeontol. Pol..

[39] Belaústegui Barahona, Z., Gibert Atienza, J. M. d., López Blanco, M. & Bajo, I. Recurrent constructional pattern of the crustacean burrow Sinusichnus sinuosus from the Paleogene and Neogene of Spain. *Acta Palaeontologica Polonica* **59**, 461–474 (2014).

[40] Hasiotis, S. *KU Ichnology: Studying the Traces of Life*, <www.https://ichnology.ku.edu/> (2014).

[41] Lourens, L. J., Hilgen, F. J., Shackleton, N. J., Laskar, J. & Wilson, D. in *Geological Time Scale* (eds F. M. Gradstein, J. G. Ogg, & A. G. Smith) Ch. 20, 409–440 (Cambridge University Press, 2004).

[42] Lourens LJ (1996). Evaluation of the Plio–Pleistocene astronomical timescale. Paleoceanography.

[43] Meulenkamp JE, Sissingh W (2003). Tertiary palaeogeography and tectonostratigraphic evolution of the Northern and Southern Peri-Tethys platforms and the intermediate domains of the African-Eurasian convergent plate boundary zone. Palaeogeogr. Palaeoclimatol. Palaeoecol..

[44] Jolivet L, Brun J-P (2010). Cenozoic geodynamic evolution of the Aegean. Int. J. Earth Sci..

[45] van Hinsbergen, D. J. & Schmid, S. M. Map view restoration of Aegean–West Anatolian accretion and extension since the Eocene. *Tectonics* **31** (2012).

[46] Hsü, K. & Montadert, L. e. a. Initial Reports of the Deep Sea Drilling Project. **42** (1978).

[47] Meulenkamp, J. E., Theodoropoulos, P. & Tsapralis, V. Remarks on the Neogene of Kythira, Greece. *Sixth Colloquium on the Geology of the Aegean Region* **1**, 355-362 (1977).

[48] van Hinsbergen DJ (2007). Discrete Plio–Pleistocene phases of tilting and counterclockwise rotation in the southeastern Aegean arc (Rhodos, Greece): Early Pliocene formation of the south Aegean left-lateral strike-slip system. J. Geol. Soc..

