# Supplementary material for: About the age and depositional depth of the sediments with reported bipedal footprints at Trachilos (NW Crete, Greece)
Source: Sci Rep. 2022 Nov 2;12:18471. doi: 10.1038/s41598-022-23296-5 (PMC9630425; doi:10.1038/s41598-022-23296-5)
Supplement: Supplementary file 1 — Supplementary Information. [file 41598_2022_23296_MOESM1_ESM.docx]

Supplementary Materials for

About the age and depositional depth of the sediments with reported bipedal footprints at Trachilos (NW Crete, Greece)

Willem Jan Zachariasse^a^ and Lucas J. Lourens^a^

^a^Department of Earth Sciences, Faculty of Geoscience, Utrecht University, Vening Meinesz building A, Princetonlaan 8a, 3584 CB, Utrecht, The Netherlands

w.j.zachariasse@uu.nl (corresponding author), l.j.lourens@uu.nl

**The Pliocene outcrop along the road to Platanos some 3 km south of the Trachilos site:**

Location has been described/sampled by Meulenkamp in 1990 (unpublished fieldnotes). The two samples (Gr 13001-13002) were used by Van Hinsbergen and Meulenkamp (2006) under the name Lar(i)das in their Table 1. No GPS coordinates are given. Outcrop is probably the same as the one visited by WJZ in 2021 at 35° 29.377N/23° 36.616E.

Lithology: beige marls and calcarenites (some very coarse and rich in bivalve debris). Thinner calcarenites with many burrows.

Planktonic foraminiferal associations: *Globoturborotalita apertura, Globigerinoides obliquus, Globigerinoides trilobus, Orbulina, Globigerina bulloides, Globigerina falconensis* and the age diagnostic species *Globorotalia puncticulata* (common), dextrally coiled *Globorotalia crassaformis* (rare), *Globorotalia scitula* (trace), and dextrally coiled *Neogloboquadrina acostaensis* (common).

Age: 3.6-3.57 Ma being the age for the FO of *Globorotalia crassaformis* and base 1e absence interval of *Globorotalia puncticulata,* respectively (see Lourens et al., 1996). This age range equates with the earliest Piacenzian (Hilgen et al., 2012).

Benthic foraminiferal associations: many shallow dwelling, predominantly epiphytic, species (also bryozoan debris). Depth marker species: *Uvigerina longistriata, Uvigerina peregrina, Planulina* *ariminensis, Gyroidina* sp., *Cibicides pseudoungerianus, Siphonina reticulata, Cibicides wuellerstorfi* (trace), *Cibicides italicus* (trace).

Depositional depth: 500-750m (see Figure 10 in Van Hinsbergen et al., 2005).

**The Messinian in the scarp some 0.6 km southwest of the church of Platanos:**

The upper part of section Platanos of Freudenthal (1969). Probably equivalent to the upper part of section Kavoussi (Frydas, 1993) which is renamed section Platanos in Frydas and Keupp (1996). The Freudenthal samples have been used by Van Hinsbergen and Meulenkamp (2006). Only samples M417-421 are still present in the Utrecht collections and used for this study.

Age diagnostic planktonic foraminifers:

M417: *Globorotalia miotumida* (trace)

M418: *Globorotalia miotumida* (common), *Globorotalia ventriosa* (rare) and dominantly left coiled *Neogloboquadrina acostaensis*

M420: *Globorotalia miotumida* (trace) and dominantly left coiled *Neogloboquadrina acostaensis*

M421: *Globorotalia miotumida* (trace). Abundant siliceous sponge spicules and presence of volcanic minerals (biotite and glass).

Age: Messinian (7.25- 6.70 Ma) for the interval with samples M417-M420. The abundant sponge spicules in sample M21 suggests a post-6.7 Ma age. Presence of volcanic minerals suggests a correlation with ash layers 4 or 5 on Gavdos which have been dated at around 6.2 Ma (see in Zachariasse and Lourens, 2021).

Benthic foraminiferal depth marker species:

M417: *Planulina ariminensis, Gyroidina sp., Cibicides pseudoungerianus, Uvigerina* *longistriata, Karreriella brady* (rare) and *Cibicides kullenbergi* (rare)

M418: *Planulina ariminensis, Gyroidina sp., Cibicides pseudoungerianus, Uvigerina* *longistriata* and traces of *Oridorsalis stellatus* and *Cibicides kullenbergi*

M419: trace occurrences of *Cibicides pseudoungerianus, Uvigerina longistriata* and *Oridorsalis stellatus.* Many epiphytes and bryozoan debris and rare rectouvigerinids. Probably sapropel.

M420: *Planulina ariminensis* (rare) and trace occurrences of *Cibicides pseudoungerianus,* *Gyroidina sp*. and *Cibicides wuellerstorfi.* Many epiphytes and bryozoan debris and rare rectouvigerinids. Poor preservation. Probably sapropel.

M421: low numbers of epiphytic species; no infaunal species. Probably sapropel

Depositional depth: 350-750m (see Figure 10 in Van Hinsbergen et al., 2005).

Full references are to be found in the main text.
